# Construction of a prognostic model for nasopharyngeal carcinoma based on serum exosomal circular RNAs and analysis of immune microenvironment

**DOI:** 10.1007/s10238-025-01920-8

**Published:** 2025-11-11

**Authors:** Hua-Jun Feng, Sai Liang, Ding-Ting Wang, Sheng-En Xu, Gang Qin

**Affiliations:** Department of Otolaryngology Head and Neck Surgery, The Affiliated Hospital of Southwest Medical University, No.25, Taiping Street, Jiangyang District, Luzhou, 646000 Sichuan China

**Keywords:** Circular RNA, Exosome, Nasopharyngeal carcinoma, Prognostic markers, Immune infiltration

## Abstract

Accumulating evidence indicates that circular RNAs (circRNAs) play a pivotal role in tumor initiation and progression. However, their expression profiles and functional roles in serum exosomes from nasopharyngeal carcinoma (NPC) remain undefined. This study aims to explore the expression patterns, biological functions, and potential prognostic significance of circRNAs in serum exosomes of NPC. CircRNA expression profiles in serum exosomes were analyzed using a circRNA microarray, along with mRNA and miRNA data from RNA sequencing and the Gene Expression Omnibus (GEO). Initially, the competitive endogenous RNA (ceRNA) regulatory network was established by integrating multiple online databases and bioinformatics tools. Subsequently, LASSO and COX regression analyses were used to construct and validate the prognostic model. Additionally, the immunological characteristics analysis was conducted using CIBERSORT. qRT-PCR was performed to further validate the expression levels of circRNAs. Finally, a total of 314 differentially expressed circRNAs were identified in NPC serum exosomes, and a ceRNA regulatory network was constructed for three of them. The prognostic risk model effectively predicts outcomes for NPC patients. Immune cell infiltration analysis revealed a significant increase in M1-type macrophages in the high-risk group, whereas the low-risk group exhibited elevated levels of resting mast cells and activated CD4 memory T cells. Additionally, immune checkpoint gene analysis revealed significantly higher expression of CD276 and ICOSLG in the high-risk group compared to the low-risk group. Our findings suggest that serum exosomal circRNAs may be promising prognostic biomarkers in NPC, warranting further functional and clinical validation.

**Supplementary Information:**

The online version contains supplementary material available at 10.1007/s10238-025-01920-8.

## Introduction

Nasopharyngeal carcinoma (NPC) is a common and highly invasive malignant tumor of the head and neck with a strong tendency for metastasis. Its global distribution exhibits clear regional and racial disparities, with the highest incidence observed in Southeast Asia, particularly in southern China [1]. Clinically, radiotherapy or concurrent chemoradiotherapy serves as the primary treatment modality for NPC. Nevertheless, approximately 30% of patients experience disease recurrence or distant metastasis following treatment, which remains the leading cause of mortality in NPC patients [2]. Currently, the identification of reliable molecular markers associated with NPC prognosis remains limited. Therefore, it is imperative to identify novel prognostic biomarkers and potential therapeutic targets for NPC.

Exosomes are extracellular vesicles ranging in size from 30 to 150 nm, secreted by most eukaryotic cells and ubiquitously present in the extracellular space [3]. Increasing evidence demonstrates that exosomes are detectable in various human body fluids, including blood, urine, saliva, and cerebrospinal fluid, and they encapsulate a diverse array of bioactive molecules, such as RNAs, DNAs, proteins, and lipids [4–6]. These molecular cargoes undergo dynamic alterations in response to pathological and physiological conditions, thereby reflecting the physiological state and functional properties of their parental cells [7]. Upon interaction with recipient cells, exosomes can be internalized via endocytic, pinocytic, or phagocytic mechanisms. Once internalized, exosomes can modulate intracellular gene expression by delivering their bioactive contents, thereby influencing cellular behaviors and contributing significantly to disease initiation and progression [8]. Accordingly, exosomes are widely recognized as key mediators of intercellular communication.

Circular RNAs (circRNAs) are a class of single-stranded, non-coding RNAs that are abundantly expressed in eukaryotic cells and generated through a process known as back-splicing [9]. Unlike canonical splicing, back-splicing connects a downstream 5' splice site (splice donor) to an upstream 3′ splice site (splice acceptor), resulting in a covalently closed circular structure stabilized by a 3′-5′ phosphodiester bond [10]. CircRNAs have diverse biological functions, including serving as miRNA sponges, protein sponges or scaffolds, modulators of immune responses, and even templates for protein translation [11]. Owing to their unique circular configuration and absence of a 5′ cap and 3′ poly(A) tail, circRNAs are resistant to exonuclease-mediated degradation. Consequently, compared to linear RNAs, circRNAs exhibit enhanced stability and reproducibility, thereby rendering them particularly pertinent to the processes of disease initiation and progression.

Growing evidence demonstrates that exosomal circRNAs can regulate multiple malignant phenotypes of tumor cells, including proliferation, invasion, migration, metastasis, metabolic reprogramming, immune evasion, and drug resistance, highlighting their critical roles in tumorigenesis and progression [12]. Li et al. first employed high-throughput sequencing to characterize the abundant and stable expression of circRNAs in serum-derived exosomes. They further observed that serum exosomal circRNAs were significantly enriched in hepatocellular carcinoma and colorectal cancer, suggesting their potential as biomarkers for distinguishing cancer patients from healthy controls [13]. Liang et al. reported that exosomal circSIPA1L3-mediated intercellular communication facilitates glucose metabolic reprogramming and promotes the progression of triple-negative breast cancer, indicating its potential as a diagnostic and prognostic biomarker as well as a therapeutic target [14]. Yu et al. identified elevated expression levels of serum exosomal circFMN2 in colorectal cancer patients, and demonstrated that its overexpression contributes to tumor progression through modulation of the miR-338-3p/MSI1 signaling axis [15]. Hu et al. found that serum exosomal circCCAR1 was upregulated in liver cancer patients and promoted tumor growth and metastasis by inducing CD8 + T cell dysfunction, thereby enhancing resistance to anti-PD-1 therapy [16]. Collectively, these findings underscore the pivotal involvement of serum exosomal circRNAs in tumor initiation and development.

Recent studies have revealed that exosomal lincRNAs and miRNAs are critically involved in the progression of NPC [17, 18]. However, the functional roles and prognostic significance of exosomal circRNAs, which demonstrate superior stability compared to linear RNAs, remain poorly understood in NPC. To address this knowledge gap, we performed a systematic analysis of circRNA expression profiles in serum exosomes from NPC patients and healthy controls, as well as mRNA expression patterns in NPC tissues and adjacent normal nasopharyngeal mucosa tissues, using circRNA microarray and transcriptome sequencing technologies. Comprehensive bioinformatics analyses, including miRNA target prediction, pathway enrichment analysis, development and validation of a prognostic model, and immune infiltration profiling, allowed us to construct a circRNA-miRNA-mRNA regulatory network associated with NPC progression and modulation of the tumor immune microenvironment. These findings contribute novel perspectives on the potential involvement of serum exosomal circRNAs in NPC pathogenesis and highlight promising candidates for prognostic biomarkers in NPC patients.

## Materials and methods

### Sample collection

Tumor tissues and normal nasopharyngeal mucosa tissues, as well as peripheral blood, were collected from patients diagnosed with NPC by pathology at the Affiliated Hospital of Southwest Medical University. Peripheral blood from healthy volunteers was also collected as a control. Informed consent was obtained from all patients, and the study was approved by the Ethics Committee of the Affiliated Hospital of Southwest Medical University.

### Serum exosome isolation

Serum exosomes were isolated by ultracentrifugation as follows: serum samples were centrifuged at 2000× *g* for 30 min at 4 °C; the supernatant was transferred to a new centrifuge tube and centrifuged at 10,000× *g* for 45 min at 4 °C to remove larger vesicles; the supernatant was filtered through a 0.22 μm filter, and the filtrate was collected; the filtrate was transferred to a new centrifuge tube and centrifuged at 100,000× *g* for 70 min at 4 °C; the supernatant was removed, and the exosomes were resuspended in 10 mL of pre-cooled 1 × PBS and centrifuged at 100,000× *g* for 70 min at 4 °C; the supernatant was removed, and the exosomes were resuspended in 250 μL of pre-cooled 1 × PBS and stored at −80 °C.

### Exosome characterization

Transmission electron microscopy (TEM): 10 μL of exosomes were taken out, and 10 μL of the sample was dropped onto a copper grid and allowed to settle for 1 min. The supernatant was absorbed with filter paper. Then, 10 μL of acetate uranyl was dropped onto the copper grid and allowed to settle for 1 min. The supernatant was absorbed with filter paper, and the sample was dried at room temperature for several minutes. Imaging was performed at 100 kV. Particle size analysis: 10 μL of exosome sample was diluted with 1 × PBS and directly used for nanoparticle tracking analysis (NTA). Western Blot: The protein concentrations of exosomes were determined using a BCA assay kit (Beyotime, China) according to the manufacturer’s instructions. Exosomes were lysed with ice-cold RIPA lysis buffer (Beyotime, China) containing a protease inhibitor cocktail (Sigma, USA). Lysates in equal amounts of proteins were separated by SDS-PAGE (Epizyme, China) and then transferred to PVDF membranes (Millipore, USA). After rinsing with TBS (Signalway Antibody, USA) several times and blocking with 5% non-fat milk (Solarbio, China), the membranes were incubated with anti-CD63 primary antibody (DLM, DL210003, China), anti-CD81 primary antibody (Proteintech, 66866-1-Ig, USA), anti-TSG101 primary antibody (DLM, DL210005, China) and anti-Calnexin primary antibody (Proteintech, 10427-2-AP, USA) overnight. Followed by thorough washing, HRP conjugated secondary antibodies (Proteintech, SA00001-1and SA00001-2, USA) were incubated with membranes in darkness for 1 h. ECL reagent (Vazyme, China) was added to the membranes to visualize the immunoreactive protein bands, and the ChemiDoc MP imaging system (BioRad, USA) was used to analyze.

### Data download

The GSE70970 and GSE102349 datasets on NPC were obtained from the Gene Expression Omnibus (GEO, https://www.ncbi.nlm.nih.gov/geo/) database. The GSE70970 dataset includes 17 normal samples and 246 tumor samples. In the dataset GSE102349, samples without survival information or with follow-up failures were excluded, and 88 samples of NPC patients were retained for survival analysis.

### CircRNA microarray

The Arraystar Human CircRNA Array V2 was used to analyze 8 serum samples (4 from NPC patients and 4 from healthy controls). Total RNA from each sample was quantified using the NanoDrop ND-1000. The sample preparation and microarray hybridization were performed based on the Arraystar’s standard protocols. Briefly, total RNAs were digested with RNase R (Epicentre, Inc.) to remove linear RNAs and enrich circular RNAs. Then, the enriched circular RNAs were amplified and transcribed into fluorescent cRNA utilizing a random priming method (Arraystar Super RNA Labeling Kit, Arraystar). The labeled cRNAs were hybridized onto the Arraystar Human CircRNA Array V2 (8 × 15 K, Arraystar). After having washed the slides, the arrays were scanned by the Agilent Scanner G2505C.

### RNA sequencing

In this study, whole-transcriptome sequencing was performed on six pairs of NPC tumor tissues and their corresponding adjacent normal tissues. The workflow is summarized as follows: Total RNA was initially extracted using TRIzol reagent (Beyotime, China) and rigorously evaluated for integrity using the Agilent 2100 Bioanalyzer (Agilent Technologies, USA), with all samples exhibiting RNA Integrity Number values greater than 7.0. For samples that met the quality criteria, rRNA depletion, RNA purification, and fragmentation were carried out in a stepwise manner, followed by the construction of strand-specific sequencing libraries. Following confirmation of library quality, paired-end sequencing was executed on the Illumina high-throughput sequencing platform (Illumina, USA).

### Differential expression analysis of circRNA, miRNA and mRNA

Based on the Arraystar Human CircRNA Array results, the edgeR package was used to conduct differential expression analysis of circRNA and mRNA. Differentially expressed circRNAs and mRNAs were screened out according to *p* < 0.05 and | Log2 (Fold Change) |> 1.5. The miRNA microarray dataset GSE70970 was downloaded from the GEO database, and then the limma package was used to conduct differential expression analysis. Differentially expressed miRNAs were screened out according to *p* < 0.05 and | Log2 (Fold Change) |> 1.5.

### Prediction of binding miRNAs of circRNAs

Based on the StarBase database and the above differentially expressed miRNAs and circRNAs, up-regulated circRNAs-down-regulated miRNAs (up_circRNA-down_miRNA) and down-regulated circRNAs-up-regulated miRNAs (down_circRNA-up_miRNA) were screened for the subsequent construction of the ceRNA network.

### Prediction of miRNA target genes

Firstly, select the differentially expressed genes of the above-mentioned mRNA and miRNA. Then, based on the miRNA-mRNA binding databases miRMap, miRanda, miRDB, TargetScan, and miTarBase, identify the miRNA-mRNA pairs. Finally, screen for the pairs of up-regulated miRNA-down-regulated mRNA (up_miRNA-down_mRNA) and down-regulated mi RNA-up-regulated m RNA (down_miRNA-up_mRNA) that exist in at least two databases simultaneously for the subsequent construction of the ceRNA network.

### CeRNA network construction

Based on the above-mentioned binding relationships between circRNA and miRNA, as well as between miRNA and mRNA, a circRNA-miRNA-mRNA regulatory network was constructed using Cytoscape v3.7.

### Construction and validation of the prognostic model

The NPC patients in the dataset GSE102349 were randomly divided into the training group and the validation group at a ratio of 1:1. Univariate Cox regression analysis was used to screen for the prognosis-related genes (*p* < 0.05). Then, LASSO regression analysis was adopted to select the characteristic genes and further construct the prognosis prediction model. The risk score was calculated based on the expression of characteristic genes and regression coefficients. Risk score = ∑(β × expr), and the NPC patients were divided into high-risk group and low-risk group based on the median of risk score. The effectiveness of the prognosis prediction model was verified by receiver operating characteristic (ROC) curve and Kaplan–Meier (KM) curve analysis.

### Functional enrichment analysis

To further investigate the functions of the target genes in the ceRNA network, Gene Ontology (GO) and Kyoto Encyclopedia of Genes and Genomes (KEGG) pathway enrichment analysis were performed using the “clusterProfiler” package, and was utilized GSEA_v4 to perform gene set enrichment analysis (GSEA) on the genes in the high-risk and low-risk groups.

### Immune infiltration analysis

Based on the risk grouping and the expression levels of various genes in cancer, immune infiltration analysis was conducted using CIBERSORT. Then, the Wilcoxon rank-sum test was used to analyze the differences in the scores of various immune cells among different groups. Additionally, we further analyzed the expression of immune checkpoint genes among different groups.

### Quantitative reverse transcription polymerase chain reaction (qRT-PCR)

The cancer tissues and adjacent normal tissues of five NPC patients were used to verify the differentially expressed circRNAs screened out. Total RNA was extracted from the tissues using the SteadyPure Quick RNA Extraction Kit from Accurate Biology (China). About 3 U/μg RNase R (Geneseed, China) was incubated with total RNA at 37 °C for 15 min to enrich circRNA. Then, cDNA synthesis was performed using the Evo M-MLV reverse transcription premix kit from Accurate Biology (China). Quantitative PCR was conducted using the SYBR Green Premix Pro Taq HS qPCR Kit from Accurate Biology (China). The relative expression levels of genes were calculated by the ΔΔCt method, and the data were analyzed by T-test. Primer sequences were as follows: hsa_circ_0036783 forward (5′-3′): AGAAGAAGTGAAAAGTTTGCCT, reverse (5′-3′): CTCCTCATATGTGAAGATCCG; hsa_circ_0011333 forward (5′-3′): GCTAGCTGAAGCCAGTTTGA, reverse (5′-3′): AGCTCACTTGTCAGCGAAA; hsa_circ_0048410 forward (5′-3′): GGGCTGGCATGGACTTTCATT, reverse (5′-3′): ATTCTCACGCATCACCGAGGA; GAPDH forward (5′-3′): AGAAGGCTGGGGCTCATTTG, reverse (5′-3′): GCAGGAGGCATTGCTGATGAT.

### Statistical analysis

Bioinformatics analysis was performed using the R language (v 4.4.2). *P* < 0.05 was considered statistically significant. The Wilcoxon rank-sum test was applied to analyze differences between two groups. An asterisk (*) was used to denote* P* < 0.05, two asterisks (**) indicated *P* < 0.01, and three asterisks (***) represented *P* < 0.001. The abbreviation “ns” was used to indicate no significant difference.

## Results

### Identification of differential circRNA, mRNA and miRNA

**Fig. 1:**
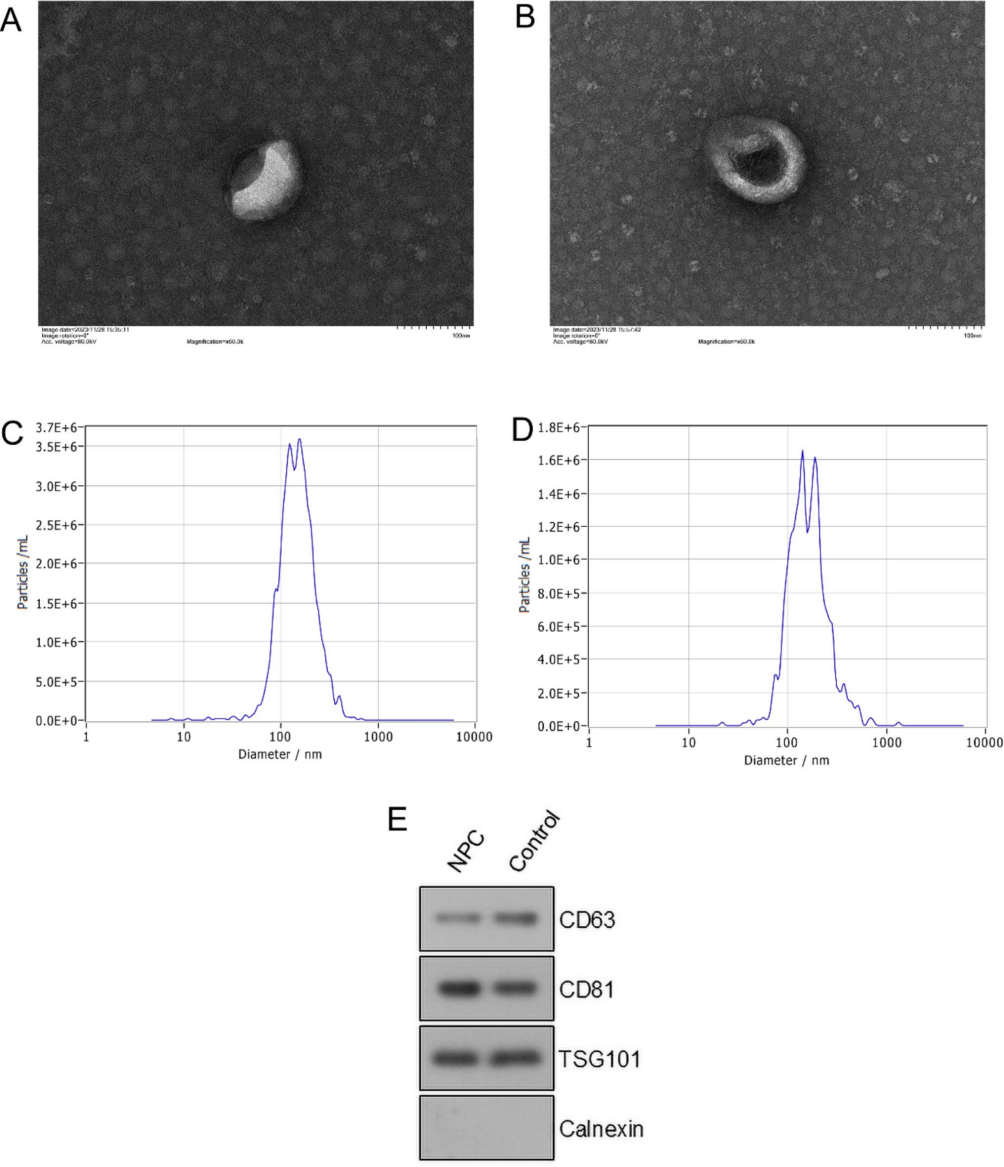
Identification of serum exosomes from NPC patients and healthy controls. **A** Representative image of serum exosomes from NPC patients under TEM. **B** Representative image of serum exosomes from healthy controls under TEM. **C** The particle size of serum exosomes from NPC patients was measured by NTA. **D** The particle size of serum exosomes from healthy controls was measured by NTA. **E** The expression levels of exosome marker proteins were detected by Western blotting

**Fig. 2:**
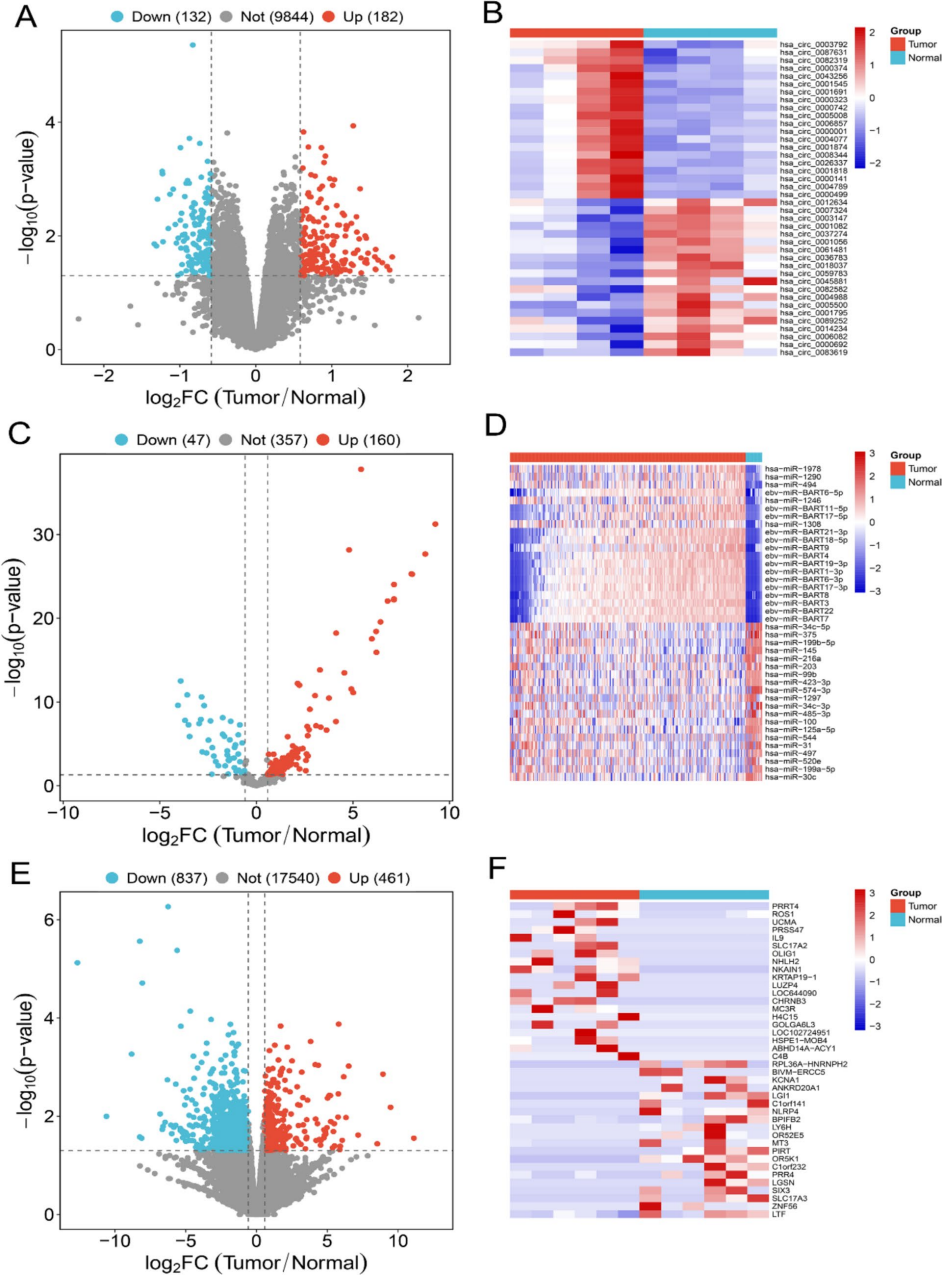
Identification of differential circRNAs, miRNAs, and mRNAs. **A** Volcano plot of differential circRNAs between NPC patients and healthy controls in serum exosomes. **B** Heatmaps of differential circRNAs between NPC patients and healthy controls in serum exosomes. **C** Volcano plot of differential miRNAs between NPC patients and normal controls in GSE70970. **D** Heatmaps of differential miRNAs between NPC patients and normal controls in GSE70970. **E** Volcano plot of differential mRNAs between Tumor tissues and normal nasopharyngeal mucosa tissues in RNA sequencing. **F** Heatmaps of differential mRNAs between Tumor tissues and normal nasopharyngeal mucosa tissues in RNA sequencing

Exosomes were isolated from serum samples using ultracentrifugation and subsequently characterized through TEM (Fig. 1A, B), NTA (Fig. 1C, D), and Western blotting (Fig. 1E). A total of 314 differentially expressed circRNAs were identified in exosomal samples obtained from individuals with NPC and healthy controls (filter criteria: log2(fold change) > 1.5, *p* < 0.05). Among these, 182 circRNAs were significantly upregulated, while 132 were significantly downregulated (Fig. 2A, B). Based on the GSE70970 dataset from the GEO database, 207 differentially expressed miRNAs were identified in NPC tissues and normal controls (filter criteria: log2(fold change) > 1.5, *p* < 0.05), with 160 miRNAs upregulated and 47 downregulated (Fig. 2C, D). Additionally, a total of 1298 differentially expressed mRNAs were detected in NPC tissues and normal nasopharyngeal mucosa tissues (filter criteria: log2(fold change) > 1.5, *p* < 0.05), including 461 mRNAs upregulated and 837 downregulated (Fig. 2E, F).

### Construction of the circRNA-miRNA-mRNA network

**Fig. 3:**
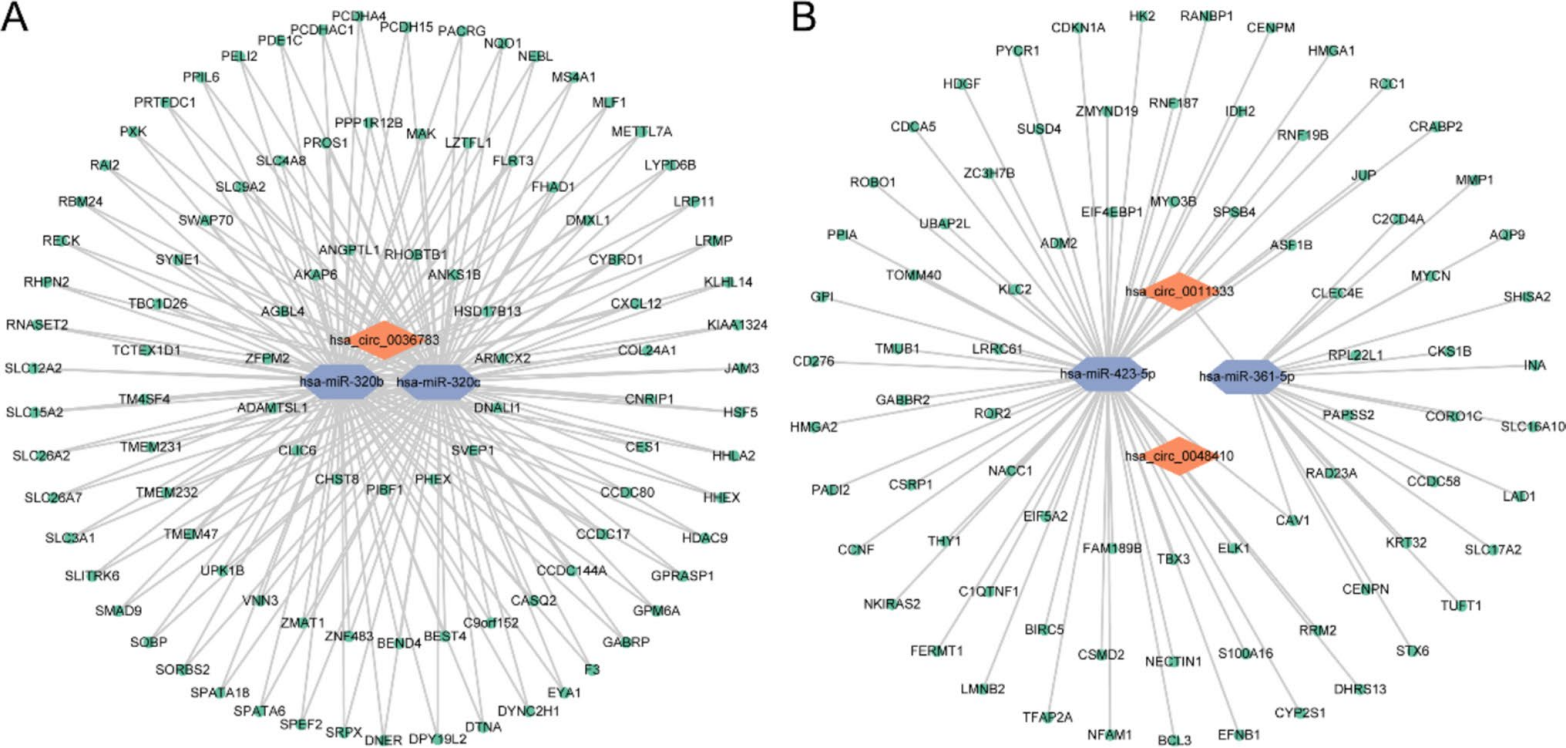
Construction of circRNA-miRNA-mRNA regulatory networks. **A** hsa_circ_0036783-miRNA-mRNA regulatory network. **B** hsa_circ_0011333-miRNA-mRNA and hsa_circ_0048410-miRNA-mRNA regulatory networks

Based on the differentially expressed genes screened out, combined with the circRNA-miRNA interactions in the Starbase database and the miRNA-mRNA interactions in the miRMap, miRanda, miRDB, TargetScan, and miTarBase databases, we successfully constructed a circRNA-miRNA-mRNA regulatory network, that includes 3 circRNAs (hsa_circ_0036783, hsa_circ_0011333, hsa_circ_0048410), 4 miRNAs (hsa-miR-320b, hsa-miR-320c, hsa-miR-361-5p, hsa-miR-423-5p) and 178 mRNAs (Fig. 3A, B).

### GO and KEGG analyses of the target genes

**Fig. 4:**
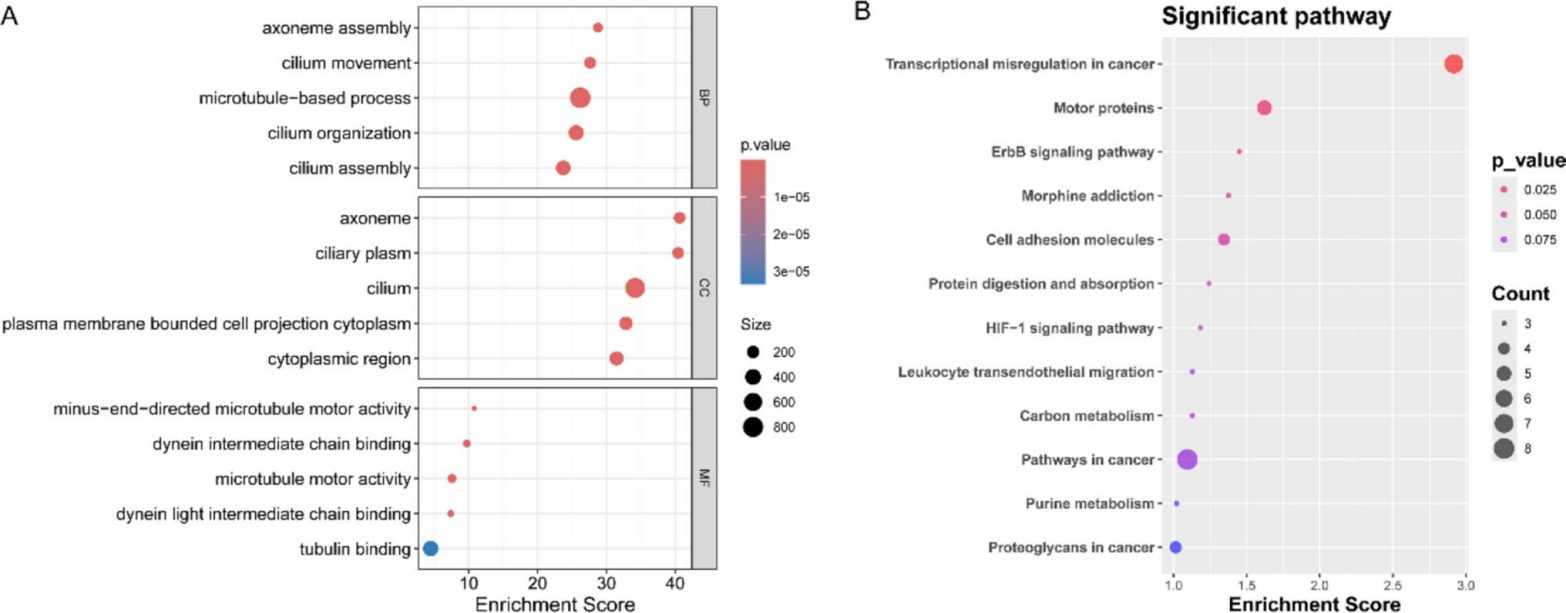
GO and KEGG analysis of target genes in the circRNA-miRNA-mRNA network. **A** GO analysis of target genes in the circRNA-miRNA-mRNA network. **B** KEGG analysis of target genes in the circRNA-miRNA-mRNA network

To further explore the functions and action pathways of mRNAs in the ceRNA network, we conducted GO and KEGG analyses. The GO analysis indicated that the target mRNAs were mainly involved in biological processes (BP) such as “anatomical structure development”, “tissue development”, and “animal organ development”, etc. The cellular component (CC) revealed that they were enriched in “cell periphery”, “plasma membrane bounded cell projection”, and “cell projection”, etc. Regarding molecular function (MF), the target genes were enriched in “protein binding”, “protein containing complex binding”, “signaling receptor binding”, etc. (Fig. 4A). KEGG analysis detected that the mRNAs were mainly enriched in signaling pathways such as “transcriptional misregulation in cancer”, “motor proteins”, “ErbB signaling pathway”, and “cell adhesion molecules”, etc. (Fig. 4B).

### Construction and validation of prognostic models

**Fig. 5:**
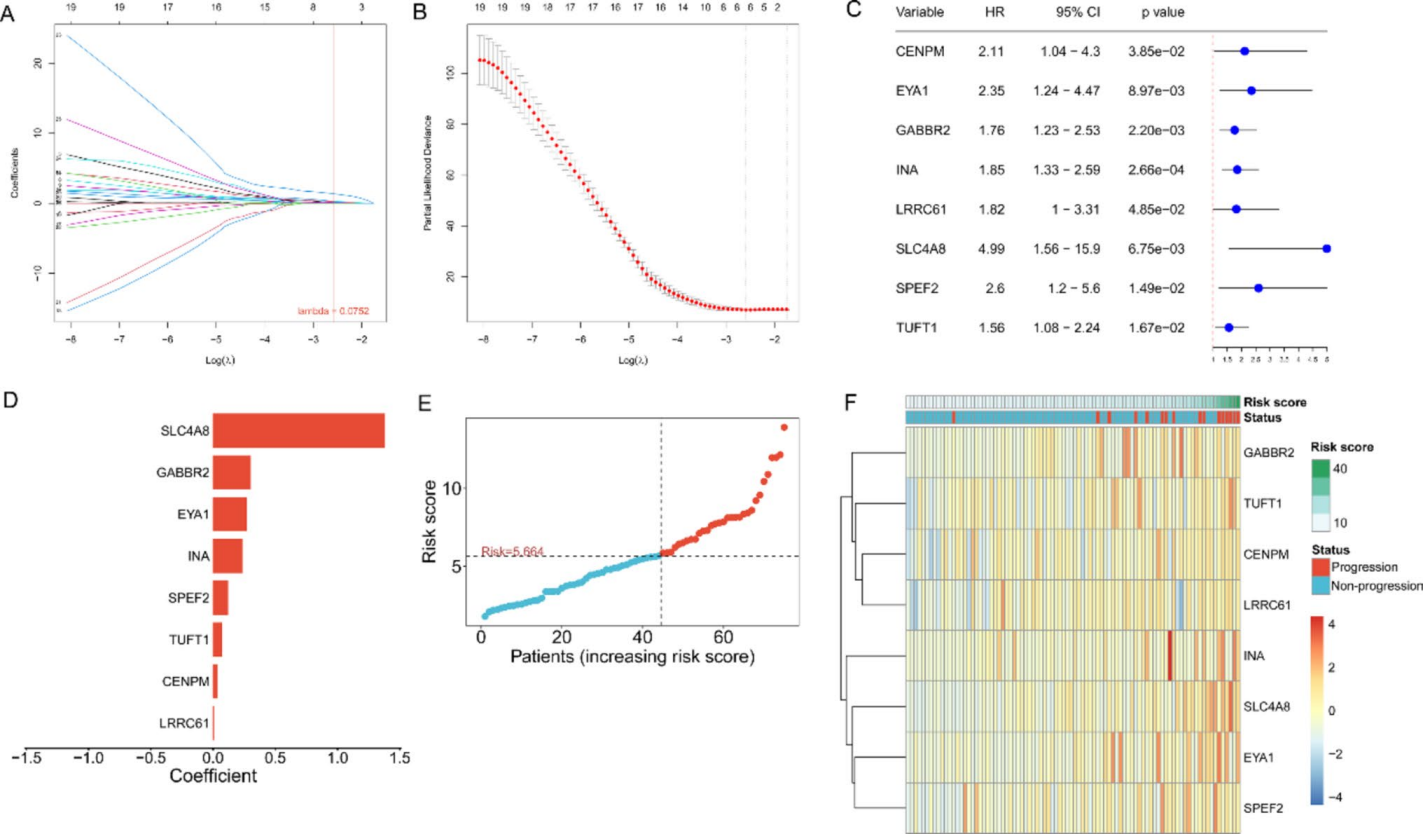
Identification of prognostic genes through univariate Cox regression and LASSO analysis. **A** LASSO coefficient profiles across different penalty levels. **B** LASSO cross-validation plot displaying the partial likelihood deviance across various values of log(λ). **C** The forest plots from univariate cox regression analyses. **D** The bar chart of the regression coefficients of genes in the model. **E** The risk scores distribution of NPC patients. **F** Heatmap of gene expression in the prognosis model

**Fig. 6:**
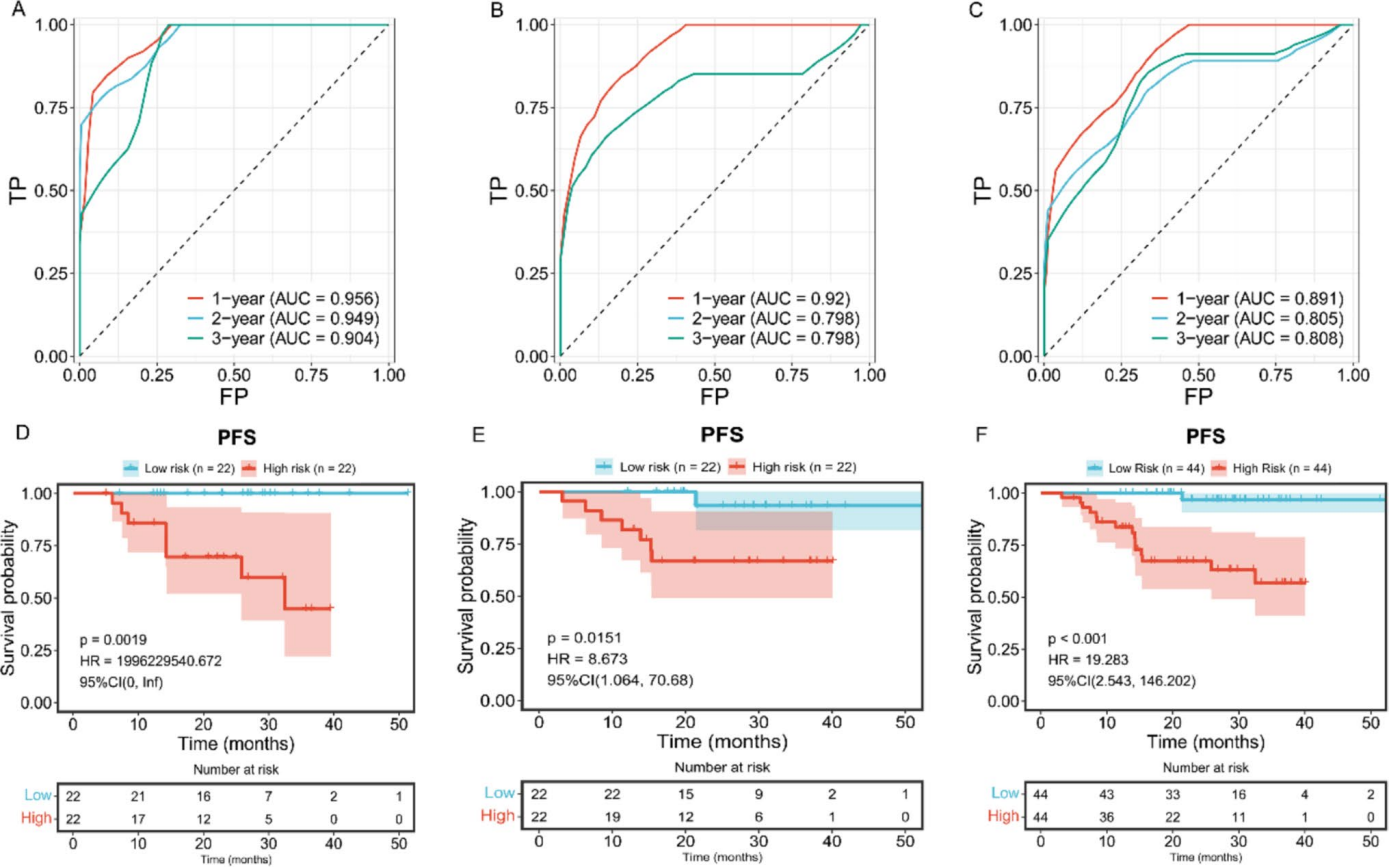
Validation of the prognostic model. **A** ROC curve analysis of training set. **B** ROC curve analysis of validation set. **C** ROC curve analysis of total set. **D** KM curve of training set. **E** KM curve of validation set. **F** KM curve of total set

**Fig. 7:**
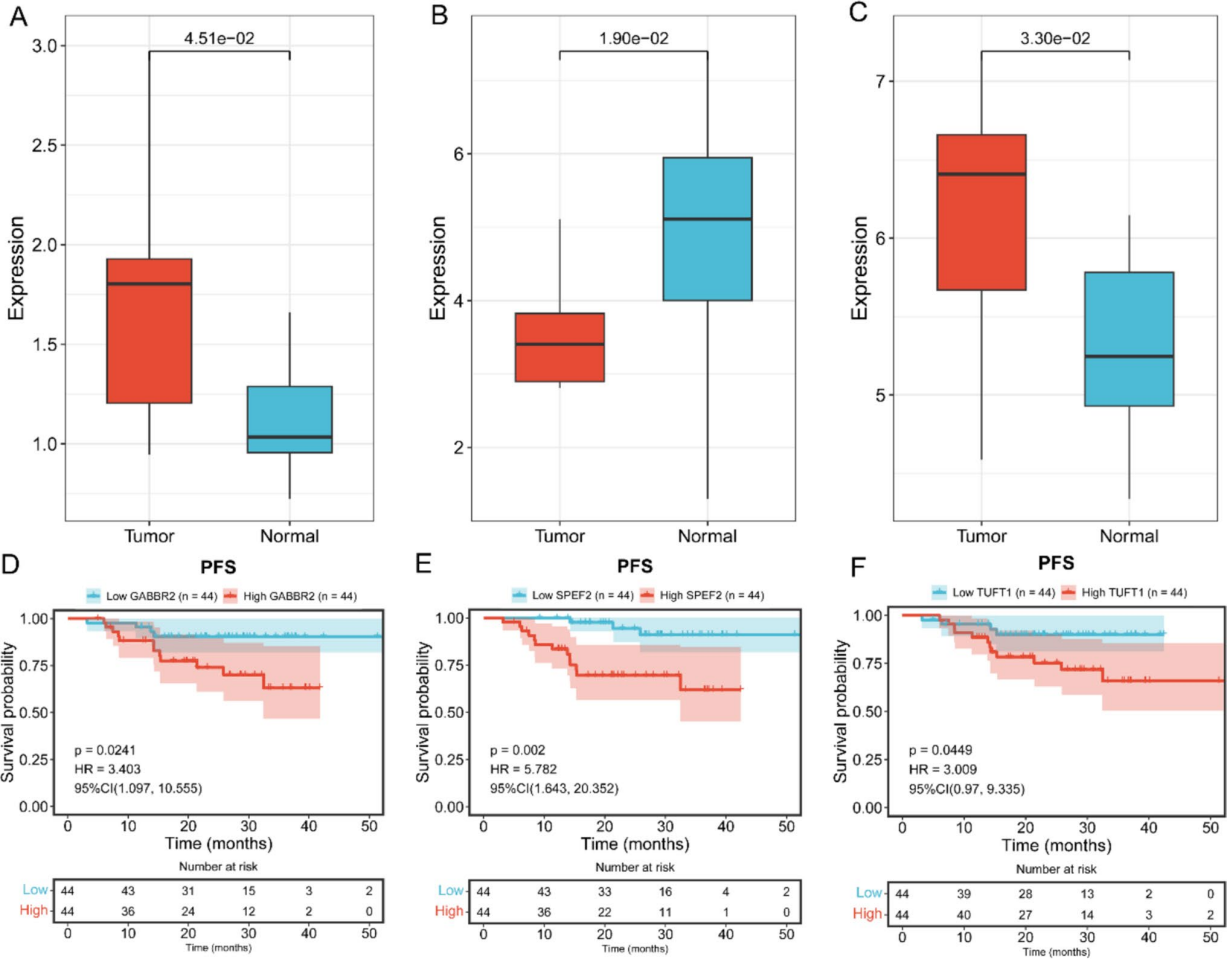
Marker genes in GSE102349 and their prognostic significance. **A** The expression levels of GABBR2 in GSE102349. **B** The expression levels of SPEF2 in GSE102349. **C** The expression levels of TUFT1 in GSE102349. **D** KM curve for GABBR2. **E** KM curve for SPEF2. **F** KM curve for TUFT1

Through univariate Cox regression analysis, with *p* < 0.05, we identified 46 genes related to prognosis (Figs.1 in supplementary information). Through LASSO regression analysis, 8 marker genes (CENPM, EYA1, GABBR2, INA, LRRC61, SLC4A8, SPEF2, TUFT1) were selected for constructing the prognostic model (Fig. 5A–C). The risk score = (0.0354 × expression level of CENPM) + (0.2655 × expression level of EYA1) + (0.2984 × expression level of GABBR2) + (0.2347 × expression level of INA) + (0.0067 × expression level of LRRC61) + (1.3783 × expression level of SLC4A8) + (0.1178 × expression level of SPEF2) + (0.0692 × expression level of TUFT1), where mRNA coefficients were calculated from multivariate Cox regression coefficients (Fig. 5D). NPC patients were divided into low-risk and high-risk groups based on the median of the risk score (Fig. 5E, F). In the training set, KM curve analysis showed a significant difference between the two groups; ROC curve was used to test the predictive accuracy of the prognostic model, with the area under the curve (AUC) for 1-year, 2-year, and 3-year PFS being 0.956, 0.949, and 0.904 respectively. Consistent results were obtained in the validation set and the total set through KM curve and ROC curve analysis, indicating that the prognostic model has high sensitivity and specificity (Fig. 6A-F). Furthermore, we further analyzed the expression of these 8 marker genes in the dataset GSE102349 and their relationship with prognosis. The results revealed that among the 8 feature genes, 3 were associated with the prognosis of NPC. Among them, GABBR2 and TUFT1 exhibited high expression levels in tumor tissues, whereas SPEF2 demonstrated low expression (Fig. 7A-F).

### GSEA enrichment analysis

**Fig. 8:**
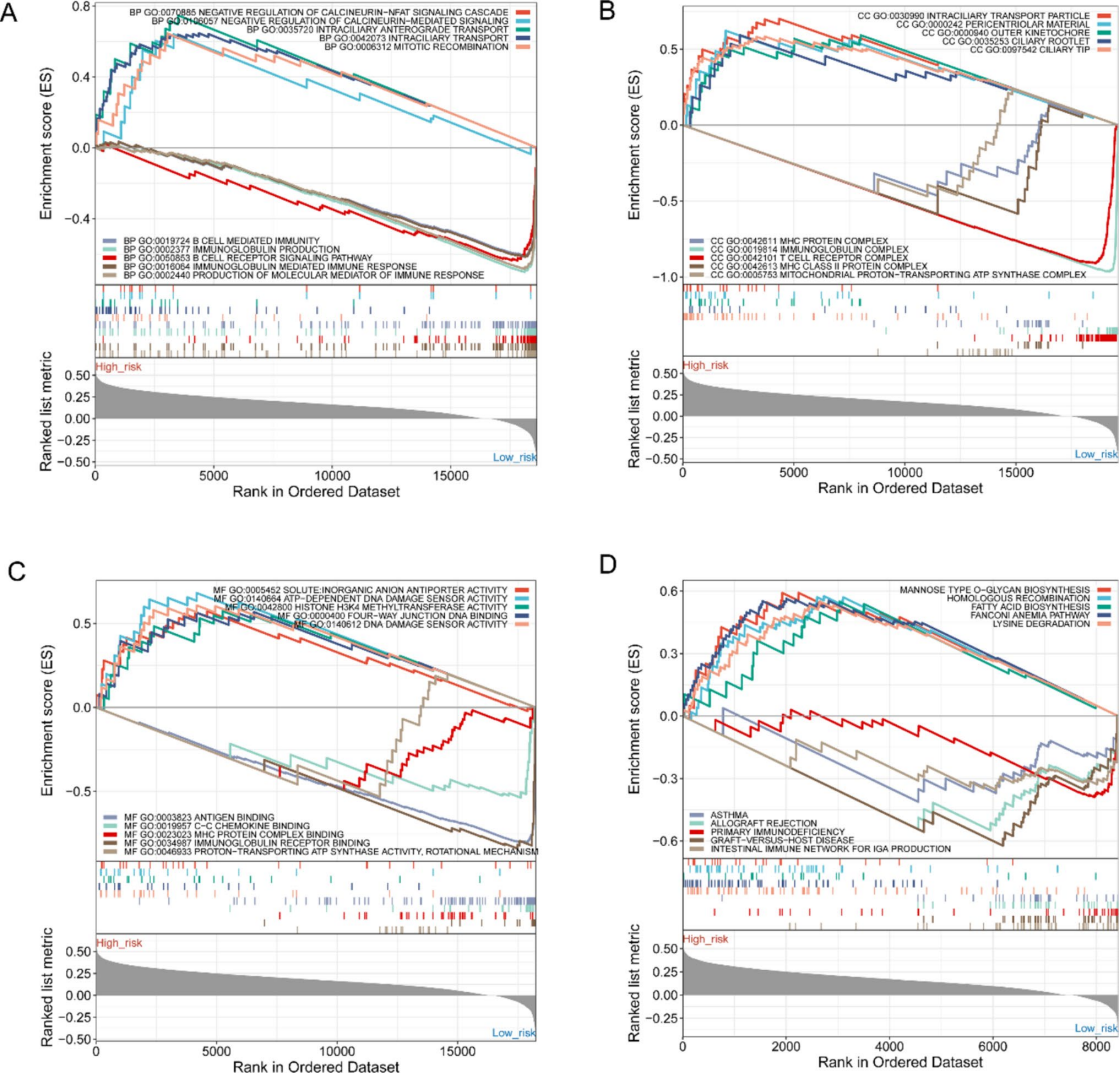
GSEA enrichment analysis in GSE102349. **A** GSEA-BP analysis in GSE102349. **B** GSEA-CC analysis in GSE102349. **C** GSEA-MF analysis in GSE102349. **D** GSEA-KEGG pathway analysis in GSE102349

GSEA enrichment analysis showed that the high-risk group was mainly associated with “asthma”, “allograft rejection”, and “primary immunodeficiency”, while the low-risk group was mainly related to “mannose type O-Glycan biosynthesis”, “homologous recombination”, and “fatty acid biosynthesis”, etc. (Fig. 8A-D).

### Immunological characteristics analysis

**Fig. 9:**
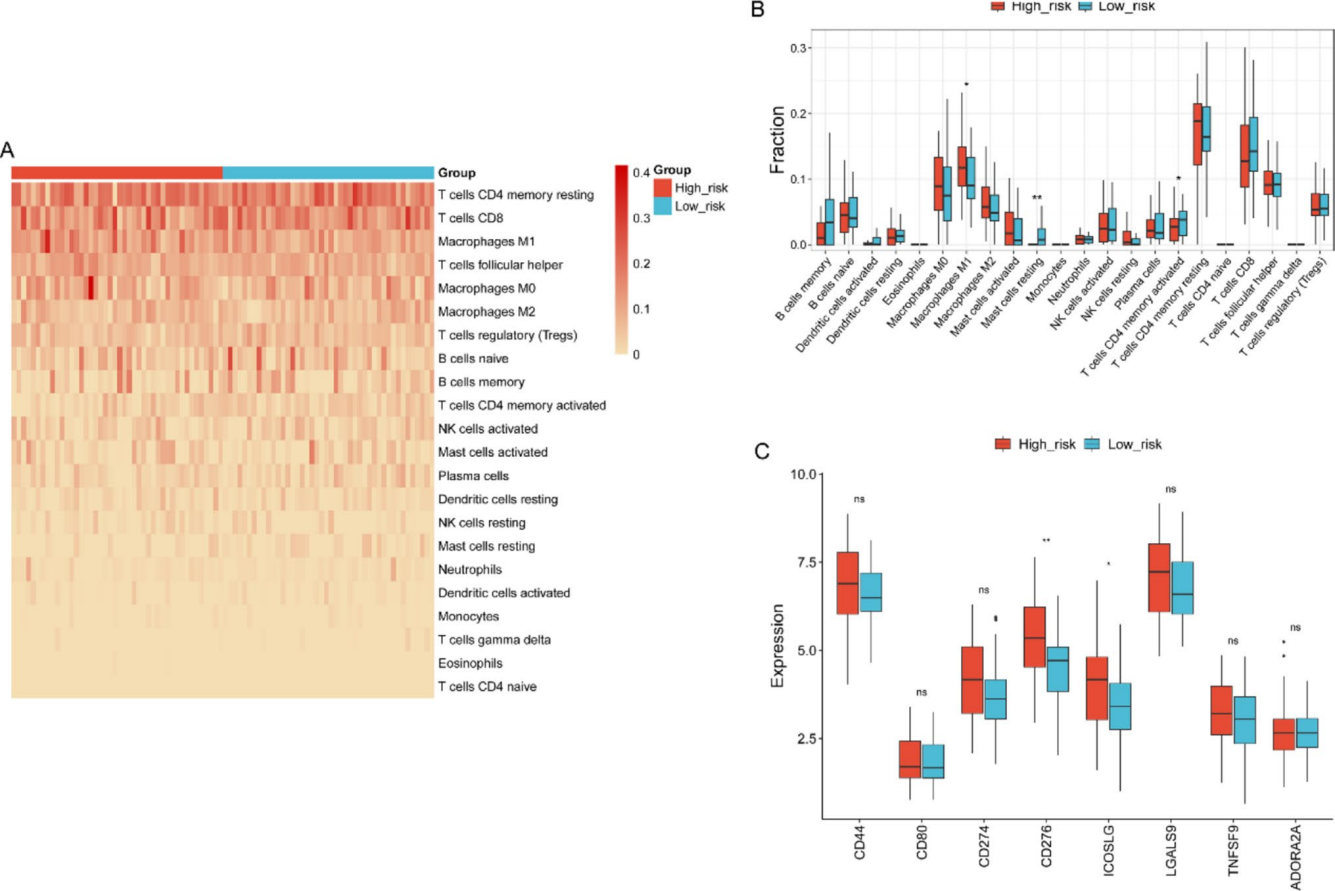
Immune cells infiltration analysis. **A** Heatmaps of immune cells infiltration level between high-risk and low-risk groups. **B** Comparative analysis of immune cell infiltration between the high-risk and low-risk groups. **C** The differential expression of immune checkpoint genes in high-risk versus low-risk groups. *P-value < 0.05, **P-value < 0.01, “ns” indicates no significant difference

Based on risk stratification, we quantitatively analyzed 22 types of immune cells to investigate the differences in immune cell composition between groups (Fig. 9A). The analysis results showed that the number of M1-type macrophages was significantly increased in the high-risk group, while resting mast cells and activated CD4 memory T cells were significantly elevated in the low-risk group (Fig. 9B). The analysis of immune checkpoint-related genes revealed that the expression of CD276 and ICOSLG was significantly higher in the high-risk group than in the low-risk group (Fig. 9C).

### qRT-PCR verification

**Fig. 10:**
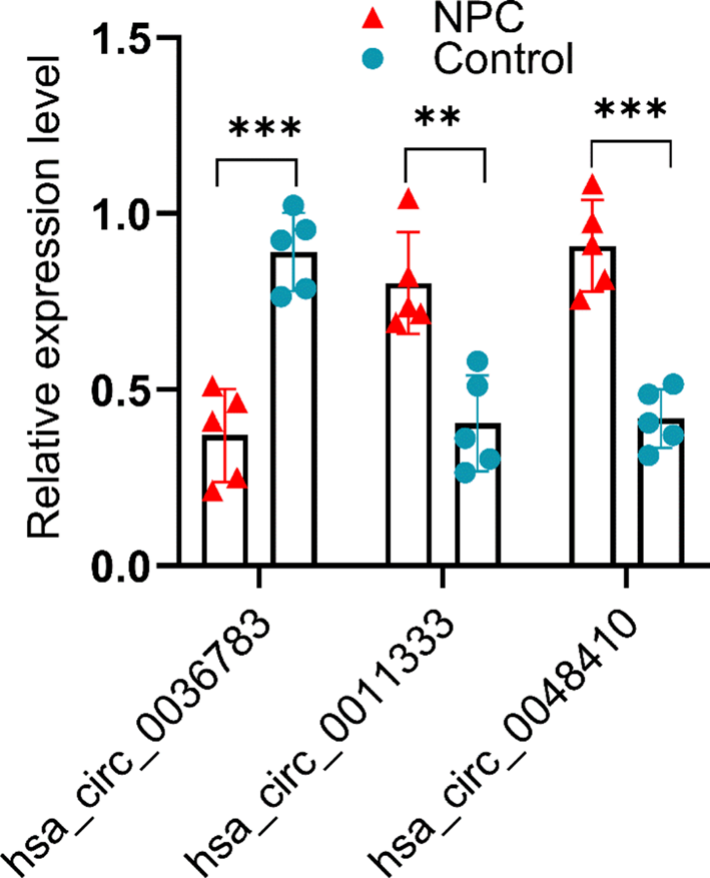
Validation of circRNA expression levels by qRT-PCR. **P-value < 0.01, ***P-value < 0.001

We selected five pairs of NPC tumors and adjacent normal tissues to verify the circRNAs in the ceRNA network by qRT-PCR. The results confirmed that hsa_circ_0011333 and hsa_circ_0048410 were upregulated in NPC, while hsa_circ_0036783 was downregulated in NPC (Fig. 10).

## Discussion

In this study, we conducted an in-depth analysis of the expression of circRNAs in NPC serum exosomes. Compared with healthy individuals, we identified 182 circRNAs significantly upregulated and 132 circRNAs significantly downregulated in NPC serum exosomes. To better understand the role of the ceRNA regulatory mechanism of circRNA in serum exosomes in NPC, we constructed a ceRNA regulatory network of circRNA in NPC serum exosomes using NPC tissue RNA-seq and GSE70970 datasets. Through the construction of the ceRNA network, we identified 3 circRNAs (hsa_circ_0036783, hsa_circ_0011333, hsa_circ_0048410), 4 miRNAs (hsa-miR-320b, hsa-miR-320c, hsa-miR-361-5p, hsa-miR-423-5p), and 178 mRNAs. KEGG analysis revealed that these genes play important roles in tumor-related signaling pathways. Additionally, through the construction and validation of a prognostic model, we screened out 8 feature genes related to the prognosis of NPC. Although only 3 out of the 8 feature genes demonstrated individual prognostic significance in univariate analysis, their combined use in a multivariate model generated a strong predictive tool, underscoring the intricate and synergistic biological mechanisms involved in tumor progression. These findings provide a research direction for the discovering of new prognostic markers and targeted therapies for NPC.

One of the important functions of circRNA is to specifically bind miRNA through its sponge effect and thereby regulate the function of target genes. A large number of studies have confirmed that the circRNA-miRNA-mRNA regulatory network plays a significant role in the progression of NPC. Hong et al. found that circCRIM1 promoted the metastasis of NPC and its resistance to docetaxel chemotherapy through the miR-422a/FOXQ1 axis [19]. Mo et al. discovered that circMAN1A2 further activated the PI3K/AKT/mTOR pathway through the miR-940/ERBB2 axis, thereby promoting the formation and progression of vascular mimicry in NPC [20]. Li et al. found that circ_0007439 inhibited the progression of NPC by regulating the miR-556-5p/PTEN axis. CircCENPM promoted the growth and stem cell characteristics of NPC cells through the miR-362-3p/BMI1 axis [21]. In addition, more studies have confirmed that circRNA can be enriched in serum exosomes and can be used as tumor-related diagnostic and prognostic markers [22–24]. Therefore, in this study, Therefore, in this study, we constructed a circRNA-miRNA-mRNA regulatory network in NPC serum exosomes, where hsa_circ_0036783, hsa_circ_0011333, and hsa_circ_0048410 play important roles in the development of NPC and are expected to become potential prognostic markers and therapeutic targets.

This study found that the three genes, GABBR2, SPEF2, and TUFT1, were significantly correlated with the progression-free survival of NPC patients in GSE102349 dataset. Moreover, GABBR2 and TUFT1 were highly expressed in NPC tissues, while SPEF2 was lowly expressed. This suggests that GABBR2 and TUFT1 may act as oncogenes in NPC, while SPEF2 may function as a tumor suppressor gene. Previous studies have confirmed that the increased expression of GABBR2 is associated with the progression of prostate cancer, cholangiocarcinoma, and malignant melanoma [25–27]. The miR-128-3p can inhibit the progression of gastric cancer by targeting TUFT1 [28]. The miR-214-3p-TUFT1 axis is closely related to liver metastasis and the maintenance of tumor stem cell characteristics in colorectal cancer [29]. The circ_0074269-miR-485-5p-TUFT1 axis can increase the resistance of cervical cancer to cisplatin by up-regulating the expression of TUFT1 [30]. CircSPEF2, which is generated by the reverse splicing of SPEF2, is lowly expressed in diffuse large B-cell lymphoma and affects its progression through the circSPEF2-miR-16-5p-BACH2 axis [31]. The above studies indicate that GABBR2, SPEF2, and TUFT1 play important roles in tumor progression. However, no relevant research reports on these genes in NPC have been found, this provides a new direction for studying NPC progression.

KEGG pathway analysis of genes associated with the serum exosomal circRNAs revealed significant enrichment in several oncologically relevant pathways, including “transcriptional misregulation in cancer,” “motor proteins,” “ErbB signaling pathway,” and “cell adhesion molecules.” While diverse, these pathways are not isolated but can be integrated into a coherent model of tumor aggression, with Notch signaling potentially serving as a central orchestrator. The Notch signaling pathway has dual roles in cancer, acting as both an oncogene and tumor suppressor, and is critically regulated by circular RNAs[32]. Thus, serum exosomal circRNAs may play a regulatory role in the Notch signaling pathway in NPC. It is well established that the immune checkpoint molecule PD-L1 plays a central role in immune escape in NPC[33]. Emerging evidence indicates that Notch3 is associated with PD-L1 and co-expressed in pancreatic sarcomatoid carcinoma, suggesting that combined targeting of PD-L1 and Notch3 may represent a promising therapeutic strategy [34]. Furthermore, this study found that in high-risk type NPC, checkpoint molecules such as CD276 and ICOSLG showed upregulated expression. Therefore, the serum exosomal circRNA-based prognostic model and its association with the immune microenvironment in NPC may reflect a broader oncological principle. This parallel implies a shared biological strategy for immune escape among histologically distinct but molecularly akin cancers. The aforementioned research findings suggest that exosomal circRNAs may function as a universal “communication medium” in intercellular signaling, coordinating key oncogenic processes across diverse tumor types. The cargo they carry does not merely reflect the state of the parent tumor but may actively participate in shaping a favorable microenvironment for progression and evasion, both locally and systemically. CircRNAs in serum exosomes are expected to serve as potential biomarkers for predicting response to immunotherapy.

Immune checkpoint inhibitors show potential in cancer treatment [35]. Previous studies have confirmed that immune cell infiltration is closely related to the progression and prognosis of NPC [36]. Immunotherapy can effectively improve the survival rate of patients with locally advanced and recurrent or metastatic NPC [37]. CD276, also known as B7 homolog 3 protein (B7H3), is a type I transmembrane protein belonging to the B7 immunoglobulin superfamily [38]. Kristmann et al. found that B7H3 can be a potential target for immunotherapy in non-small cell lung cancer [39]. Chen et al. discovered that EBV inhibits NK cell-mediated anti-tumor immunity by up-regulating the expression of B7H3 [40]. Ma et al. further confirmed through clinical experiments that the antibody–drug conjugate YL201 targeting B7H3 can improve the clinical efficacy of patients with recurrent and metastatic NPC [41]. ICOSLG belongs to the B7-CD28 immunoglobulin superfamily and is a ligand of ICOS. Zhang et al. found that high expression of ICOSLG is significantly associated with the overall survival rate of NPC patients [42]. Our research found that the expression of immune checkpoint genes B7H3 and ICOSLG was significantly increased in NPC tissues of high-risk group, indicating the complexity of multi-gene interactions in the immune microenvironment of NPC. The specific molecular mechanism remains to be further explored.

Immunological characteristics analysis revealed that the abundance of M1-type macrophages was significantly higher in the high-risk group. The paradoxical observation that a high M1 macrophage signature is associated with poor prognosis in our high-risk NPC cohort can be explained by recent advancements in our understanding of tumor-associated macrophage (TAM) biology, which moves beyond the simplistic M1/M2 dichotomy [43]. Firstly, it is now well-established that TAMs can exist in a “dysfunctional” or “exhausted” state. For instance, several studies have demonstrated that TAMs expressing TREM2 exhibit impaired phagocytic function and create an immunosuppressive niche, despite potentially expressing certain inflammatory genes [44, 45]. The metabolic environment of aggressive tumors could further induce such a dysfunctional state in M1-like cells [46]. Secondly, high-dimensional analyses have revealed that TAMs frequently adopt mixed phenotypes. Pan-cancer single-cell studies and research specific to head and neck cancers have identified macrophage populations co-expressing both M1 and M2 markers, which may drive tumor progression through distinct mechanisms like matrix remodeling and sustained inflammation [47, 48]. Therefore, we speculate that the M1-high signature in our study likely represents either a dysfunctional state or a pro-tumorigenic hybrid macrophage population, rather than effector anti-tumor macrophages. This aligns with the broader concept that chronic inflammation, even involving “classically” associated signals, can fuel cancer progression [49]. Future studies with single-cell resolution will be crucial to precisely define this population in NPC.

Our study has several limitations that warrant consideration. Firstly, the prognostic model was developed and validated in a relatively small cohort, which inherently increases the risk of overfitting and limits statistical power. Although we employed bootstrap internal validation to address this, the findings require further validation in larger, independent external datasets once they become available. Secondly, although we analyzed the expression of circRNAs in serum exosomes from NPC patients, the expression levels of circRNAs, miRNAs, and mRNAs in both tissue and serum exosome samples may differ, necessitating validation in larger and more homogeneous cohorts. Furthermore, although comprehensive bioinformatics analysis was performed, future studies must incorporate functional experiments to rigorously validate the biological roles of these circRNAs and elucidate their underlying molecular mechanisms.

In conclusion, our study suggests a potential link between serum exosomal circRNA signatures and both prognosis and immune infiltration in NPC. While further validation is needed, these findings provide a preliminary basis for non-invasive biomarker development and highlight the importance of exploring the underlying biological mechanisms.

## Supplementary Information

Below is the link to the electronic supplementary material.

Supplementary file1 (TIF 3014 KB)

## Data Availability

The data and materials in the current study are available from the corresponding author on reasonable request.
