# Supplementary figures and images for: Construction of a prognostic model for nasopharyngeal carcinoma based on serum exosomal circular RNAs and analysis of immune microenvironment
Source: Clin Exp Med. 2025 Nov 11;25(1):370. doi: 10.1007/s10238-025-01920-8 (PMC12605549; doi:10.1007/s10238-025-01920-8)

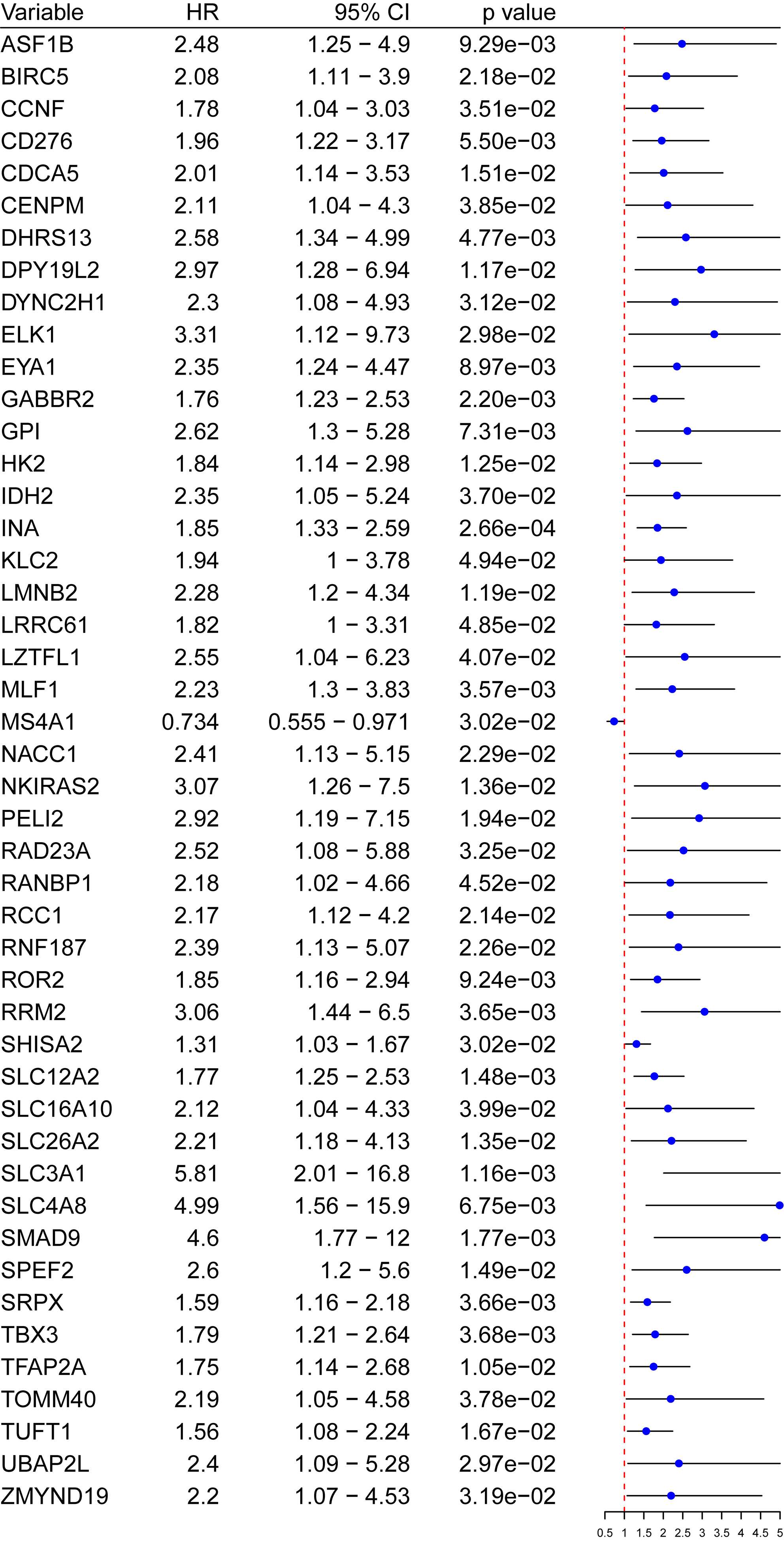

Supplement: Supplementary file 1 — Supplementary file1 (TIF 3014 KB) [file 10238_2025_1920_MOESM1_ESM.tif]
